# Psychometric validation of a culturally adapted health belief model scale for breast cancer screening in Chinese women

**DOI:** 10.1371/journal.pone.0331279

**Published:** 2025-09-03

**Authors:** Yang Liao, Suhaily Mohd Hairon, Najib Majdi Yaacob, Tengku Alina Tengku Ismail, Li Luo

**Affiliations:** 1 Department of Community Medicine, School of Medical Sciences, Universiti Sains Malaysia, Kubang Kerian, Kelantan, Malaysia; 2 The Second Affiliated Hospital of Guizhou University of Traditional Chinese Medicine, Guiyang, Guizhou, China; 3 Biostatistics and Research Methodology Unit, School of Medical Sciences, Universiti Sains Malaysia, Kubang Kerian, Kelantan, Malaysia; 4 Department of Oncology, Guihang Guiyang Hospital, Guiyang, Guizhou, China; South China Normal University, CHINA

## Abstract

This study aimed to validate a culturally adapted Health Belief Model (HBM) questionnaire for assessing breast cancer screening beliefs among Chinese women and to evaluate its structural validity and measurement invariance. A cross-sectional survey was conducted with 314 women aged 18–70 in Guizhou Province, China, using multistage sampling. The original HBM questionnaire underwent cultural adaptation and expert content review. Exploratory and confirmatory factor analyses were conducted to examine the factor structure, and measurement invariance was tested across residential settings and age groups. A seven-factor solution was identified, encompassing all six core HBM constructs, with self-efficacy splitting into two empirically distinct dimensions. The final 41-item model demonstrated strong model fit (CFI = 0.914, TLI = 0.906, RMSEA = 0.059, SRMR = 0.060), high internal consistency (ω ≥ 0.88), and full invariance across subgroups. Convergent validity and inter-construct correlations aligned with theoretical expectations. In particular, strong positive associations were observed among self-efficacy, perceived benefits, and cues to action, while perceived barriers showed inverse relationships with perceived severity and risk perception. These findings offer empirical justification for both single-construct and multi-construct intervention strategies. The validated scale provides a psychometrically sound and culturally grounded tool for identifying psychological barriers to screening among Chinese women. It may guide the design of theory-informed public health interventions that address individual belief profiles and promote screening uptake.

## Introduction

Breast cancer remains the leading cause of cancer-related death among women [1]. Although Breast Cancer Screening (BCS) is an effective strategy for reducing mortality, participation rates remain suboptimal in many low- and middle-income countries. In China, screening coverage is estimated to be below 25%, especially in rural areas [2,3]. Low participation rate for BCS delays early detection and increases the burden on healthcare systems [4]. A better understanding of the behavioral factors influencing BCS is crucial for developing interventions and public health strategies [5].

The Health Belief Model (HBM) is a well-established framework used for explaining health behaviors, including behaviors related to cancer screening [6]. It comprises six core constructs: perceived susceptibility, perceived severity, perceived benefits, perceived barriers, self-efficacy, and cues to action [7]. Collectively, these constructs predict an individual’s likelihood of engaging in health behaviors by evaluating perceived health threats and balancing preventive actions’ perceived advantages and obstacles.

Most studies utilized HBM without validating it [8]. In China, few studies have applied rigorous psychometric techniques, such as exploratory and confirmatory factor analyses, to test the structural validity of HBM tools [9]. This methodological gap limits a comprehensive understanding of the model’s applicability across cultural contexts [10]. Cultural factors such as traditional beliefs, and gender roles may influence how Chinese women interpret and respond to HBM constructs, underscoring the importance of cross-cultural validation for HBM [9].

This study aims to validate a HBM questionnaire adapted for Chinese women. The three goals of this study are: 1) assess the structural validity of the adapted questionnaire through Exploratory Factor Analysis (EFA) and Confirmatory Factor Analysis (CFA); 2) examine the cross-cultural relevance and theoretical consistency of the six core Health Belief Model constructs among Chinese women; 3) test measurement invariance (MI) across sociodemographic subgroups, including residential settings and age groups.

This study contributes to a deeper understanding of BCS behaviors in China and provides empirical support for developing culturally sensitive health education strategies [11]. It achieves this by bridging theoretical frameworks and practical applications by systematically validating the HBM within a Chinese sociocultural context. The findings offer healthcare professionals and policymakers a reliable tool for guiding BCS interventions, while also contributing to methodological advancements in cross-cultural health behavior research.

## Materials and methods

### Study design and participants

This cross-sectional study used a multistage sampling approach to enhance sample representativeness and reduce selection bias. Participants were recruited from two demographically diverse cities in Guizhou Province, China: Guiyang, the provincial capital, and Zunyi, a prefecture-level city. These cities were selected not only based on urban–rural differences, socioeconomic status, and accessibility to breast cancer screening services but also to capture variations in economic development and healthcare resource allocation. By selecting one provincial capital and one regular prefecture-level city, the study encompasses a broader spectrum of healthcare accessibility and social diversity, thereby enhancing the representativeness and generalizability of the findings. In each city, four districts were randomly selected, followed by the random selection of communities within those districts.

Participants were eligible: 1) Women aged 18–70 years; 2) Residents of Guizhou Province for ≥1 year; 3) Chinese citizens.

A total of 314 participants completed the survey. Responses from ineligible participants or those with incomplete data were excluded from the final analysis. The sample size was determined based on established recommendations for factor analysis. A minimum of 300 participants is generally considered sufficient to achieve stable factor solutions and adequate power for both exploratory and confirmatory factor analysis [12].

### Instrument development and measures

The BCS instrument used in this study was adapted from a validated HBM questionnaire developed by Malaysian researchers [13], originally in English. The version used in this study comprises 61 items across the six core HBM constructs: perceived susceptibility, severity, benefits, barriers, self-efficacy, and cues to action. A rigorous cross-cultural adaptation process was conducted following internationally recognized principles proposed by Wild et al. (2005), including forward translation into Chinese by bilingual public health professionals and independent back-translation by another bilingual team [14]. The forward translation was carried out by two bilingual public health experts, followed by a reconciliation step to address any discrepancies. Discrepancies were resolved by consensus to ensure semantic and theoretical consistency [15]. The back-translation was then reviewed by another bilingual team, and differences were addressed to ensure both linguistic and cultural appropriateness. After translation, a multidisciplinary panel of five experts in public health, behavioral science, and oncology was invited to systematically evaluate the content validity of each item. The evaluation covered clarity, relevance, representativeness, and necessity. Based on expert feedback, several items were revised to enhance theoretical and contextual relevance. We calculated Item-Level Content Validity Index (I-CVI), Content Validity Ratio (CVR), and modified Kappa coefficients to quantitatively assess expert agreement. Furthermore, 10 native Chinese speakers from the target population participated in a structured face validity assessment. Each item was rated for clarity and comprehensibility, and item-level face validity indices were computed. Minor modifications were made to improve item wording and readability. The finalized Chinese version of the questionnaire was subsequently subjected to psychometric validation using factor analysis techniques. The English version of the final adapted questionnaire is available in S1 File.

### Data collection procedure

Data were collected between August and October 2024 through a self-administered online questionnaire. Participants were recruited via community health centers, social media, and university networks to ensure diversity. The survey was hosted on a secure third-party platform, accessible via WeChat login and a QR code.

On the first page of the questionnaire, participants were provided with essential information regarding the study’s aims, their rights as participants, and data confidentiality. Proceeding to complete the questionnaire was considered as providing implied informed consent.

### Statistical analysis

#### Descriptive statistics.

Descriptive statistics were performed using IBM SPSS Statistics version 26.0 to summarize participants’ demographics and responses to the HBM questionnaire. Means and standard deviations were calculated for continuous variables, and frequencies and percentages for categorical variables. EFA, CFA, and measurement invariance analyses were conducted using R version 4.4.2 (R Core Team, Vienna, Austria) within RStudio (Posit, Boston, MA) with the lavaan [16] and semTools packages to explore factor structure, assess construct validity, and evaluate cross-group model stability.

A conceptual path diagram of the final CFA model was manually developed in IBM SPSS Amos 28.0 to illustrate the relationships between latent constructs and observed indicators.

#### Data analysis.

Initially, CFA was conducted based on the assumption that the original instrument had a stable and established factor structure. However, the initial CFA model produced over 20 large modification indices (MIs), suggesting substantial model misfit and potential structural instability. Given the extent of required modifications, EFA was subsequently conducted on the same dataset to identify the latent structure within the current sample. A seven-factor solution emerged, which informed the revised CFA model. While using separate samples for EFA and CFA is ideal, conducting both analyses on the same dataset is widely accepted in initial validation studies, especially when adapting existing instruments for new populations or cultural contexts. This approach can still produce robust and interpretable results [17].

#### Exploratory Factor Analysis (EFA).

EFA was conducted to examine the underlying structure of the culturally adapted HBM questionnaire. The minimum residual (minres) extraction method was applied with oblimin rotation [18], allowing for correlation among factors, in line with the theoretical assumptions of the HBM. The following thresholds were applied: KMO ≥ 0.80 and a significant Bartlett’s test of sphericity (p < 0.001) to confirm sampling adequacy, factor loadings (FLs) and communalities (h^2^) ≥ 0.50, and Cronbach’s alpha ≥ 0.70 [18]. The final factor solution was selected based on empirical performance and conceptual relevance.

To further strengthen structural validation and ensure cross-method robustness, supplementary analyses were performed. Exploratory Graph Analysis (EGA) was conducted using the EGAnet package in R to examine the network-based dimensional structure of the retained items. The graphical least absolute shrinkage and selection operator (glasso) model was applied to estimate the regularized partial correlation network. A visual network plot was generated to support dimensional inspection.

In addition, Item Response Theory (IRT) analysis was conducted using a graded response model (GRM) via the mirt package in R. The model estimated item discrimination (a) and difficulty (b) parameters for each retained item. The Test Information Function (TIF) was used to evaluate measurement precision across the latent trait continuum.

#### Confirmatory Factor Analysis (CFA).

CFA was used to validate the factor structure identified in EFA. The analysis was performed using the robust maximum likelihood estimator (MLR), which adjusts for non-normality [19]. Multiple models were tested based on varying item retention criteria. The final model was chosen based on model fit, parsimony, and theoretical interpretability. Model fit was evaluated based on the following criteria:1) Comparative Fit Index (CFI) ≥ 0.90; 2) Tucker–Lewis Index (TLI) ≥ 0.90; 3) Root Mean Square Error of Approximation (RMSEA) ≤ 0.08; 4) Standardized Root Mean Square Residual (SRMR) ≤ 0.08 [19]. To further improve model fit, five residual covariances were added based on modification indices (MIs), each supported by theoretical justification [19]. Internal consistency was assessed using McDonald’s omega (ω), with values ≥ 0.70 considered acceptable [20]. Convergent validity was evaluated using composite reliability (CR) and average variance extracted (AVE), with thresholds of ≥ 0.70 for CR and ≥ 0.50 for AVE [18].

#### Measurement Invariance Analysis (MI).

MI was assessed across two demographic variables: residential setting and age group. To ensure adequate group sizes and facilitate meaningful comparisons, residential areas were recoded into two categories. The “urban” category included participants originally coded as “city,” while the “non-urban” category combined “suburban” and “rural” areas. Age was grouped into 18–40 and 41–70 years, based on sample distribution and life-stage relevance. Multi-group confirmatory factor analysis (MG-CFA) was conducted using a stepwise procedure to test four hierarchical levels of invariance: configural, metric, scalar, and strict [21]. Configural invariance assessed the applicability of the same factor structure across groups [22]. Metric invariance constrained factor loadings, scalar invariance added constraints on item intercepts, and strict invariance further constrained residual variances [21]. Model fit was evaluated using the CFI, TLI, RMSEA, and SRMR. Invariance was determined based on changes in model fit between nested models, with ΔCFI ≤ 0.010, ΔRMSEA ≤ 0.015, and ΔSRMR ≤ 0.03 indicating acceptable invariance [22].

### Ethics statement

This study was approved by the Human Research Ethics Committee of Universiti Sains Malaysia (JEPeM Code: USM/JEPeM/KK/24010004). All procedures involving human participants were conducted in accordance with the ethical principles outlined in the Declaration of Helsinki.

Participants were provided with detailed study information, including the research purpose, procedures, and data confidentiality, on the first page of the online questionnaire. Participation was entirely voluntary. Although no formal written or verbal consent was obtained, implied informed consent was assumed based on participants’ decision to proceed with the survey after reading the provided information. This consent procedure was reviewed and approved by the ethics committee.

Data were collected through an anonymous online questionnaire platform. Although IP addresses were automatically recorded by the system for basic traffic management, the research team did not download, access, or use any IP data in the analysis. No personal identifiers were collected, and all responses were handled in an anonymized and de-identified manner.

The study did not involve any clinical intervention or access to hospital settings. It was a non-clinical, minimal-risk behavioral research project conducted by a registered research student at Universiti Sains Malaysia. Therefore, no additional ethics approval from Chinese institutions was required. The study did not involve minors, and no consent from parents or guardians was necessary.

## Result

### Participant characteristics

A total of 314 women participated in this study, with a mean age of 35.34 years (SD = 11.68, range: 18–65 years). The majority resided in urban areas (70.4%), held at least a bachelor’s degree (63.7%), and were employed (54.8%). Most participants were married (56.4%), and 89.5% reported no chronic diseases. Given that measurement invariance was tested across residential areas, the urban–rural distribution provided a meaningful basis for group comparisons. A detailed summary of participant characteristics is presented in Table 1.

**Table 1:** Participant characteristics.

Socio-demographic Characteristic	n (%)
**Age (years)**	
Mean (SD)	35.34(11.68)
Range	18–65
**Age Groups**	
18–25	88(28%)
26–35	78(24.8%)
36–45	80(25.5%)
46–55	56(17.8%)
56–65	12(3.8%)
**Residence**	
Urban	221(70.4%)
Suburban	42(13.2%)
Rural	51(16.2%)
**Education**	
Primary school or below	2(0.6%)
Junior high school	15(4.8%)
High school/vocational school	36(11.5%)
Associate degree	61(19.4%)
Bachelor’s degree	181(57.6%)
Master’s degree or above	19(6.1%)
**Employment Status**	
Employed	172(54.8%)
Student	75(23.9%)
Unemployed/Retired	67(21.3%)
**Household Income (RMB per month)**	
**Median(IQR)**	**3646(1707-5500)**
Below 2000	93(29.6%)
2001--4000	81(25.8%)
4001--6000	83(26.4%)
6001--8000	36(11.5%)
8001-10000	14(4.5%)
Above 10001	7(2.2%)
**Family Member**	
1 person	6(1.9%)
2 people	30(9.6%)
3-4 people	173(55.1%)
5 people and above	105(33.4%)
**Housing Situation**	
Own house(including living with parents)	281(89.8%)
Renting	32(10.2%)
**Chronic Diseases**	
Yes	33(10.5%)
No	281(89.5%)
**Medical Payment**	
Urban public basic medical insurance	211(67.2%)
Urban resident basic medical insurance	87(27.7%)
Fully self-funded	16(5.1%)
**Family History of Breast Cancer**	
Yes	12(3.8%)
No	302(96.2%)
**Marital Status**	
Single	112(35.7%)
Married	177(56.4%)
Divorced/Widowed	25(7.9%)

### Exploratory Factor Analysis (EFA)

EFA supported a seven-factor structure, reflecting the original six HBM domains with an empirical split in the self-efficacy construct into two conceptually coherent subdimensions. The KMO measure was 0.88 and Bartlett’s test of sphericity was significant (χ^2^ = 14,963.75, df = 1830, p < 0.001), indicating data adequacy. Three item retention strategies were explored in this study. Option 1: Items with FLs > 0.50 and h^2^ > 0.45; Option 2: Items with FLs and h^2^ ≥ 0.50; Option 3: Same as Option 2, but retaining item Q10 due to theoretical importance.

Option 3 was adopted. A total of 20 items were removed due to low loadings, weak communalities, or cross-loadings. The final solution included 41 items across seven factors, with standardized loadings ranging from 0.480 to 0.934 (Table 2). The seven extracted factors collectively accounted for 65.5% of the total variance, with individual factor contributions ranging from 5.6% to 13.3% (Table 3). The RMSR was 0.028, and Cronbach’s alpha values for the extracted factors ranged from 0.88 to 0.94, demonstrating strong internal consistency (Table 4). These results support the robustness and theoretical consistency of the seven-factor structure within the HBM framework.

**Table 2:** Standardized factor loadings from exploratory factor analysis (EFA).

Item	Perceived Severity	Perceived Susceptibility	Perceived Benefits	Perceived Barrier	Self-Efficacy 1	Self-Efficacy2	Cues To Action
**Q1**	0.772	—	—	—	—	—	—
**Q2**	0.901	—	—	—	—	—	—
**Q3**	0.832	—	—	—	—	—	—
**Q6**	—	0.849	—	—	—	—	—
**Q7**	—	0.872	—	—	—	—	—
**Q8**	—	0.934	—	—	—	—	—
**Q9**	—	0.868	—	—	—	—	—
**Q10**	—	—	0.499	—	—	—	—
**Q14**	—	—	0.813	—	—	—	—
**Q15**	—	—	0.819	—	—	—	—
**Q16**	—	—	0.804	—	—	—	—
**Q17**	—	—	0.731	—	—	—	—
**Q18**	—	—	0.864	—	—	—	—
**Q19**	—	—	0.726	—	—	—	—
**Q21**	—	—	0.706	—	—	—	—
**Q31**	—	—	—	0.765	—	—	—
**Q32**	—	—	—	0.815	—	—	—
**Q33**	—	—	—	0.796	—	—	—
**Q34**	—	—	—	0.894	—	—	—
**Q35**	—	—	—	0.879	—	—	—
**Q36**	—	—	—	0.870	—	—	—
**Q37**	—	—	—	0.707	—	—	—
**Q38**	—	—	—	0.669	—	—	—
**Q41**	—	—	—	—	0.750	—	—
**Q42**	—	—	—	—	0.795	—	—
**Q43**	—	—	—	—	0.794	—	—
**Q44**	—	—	—	—	0.696	—	—
**Q45**	—	—	—	—	0.709	—	—
**Q48**	—	—	—	—	—	0.480	—
**Q50**	—	—	—	—	—	0.861	—
**Q51**	—	—	—	—	—	0.921	—
**Q52**	—	—	—	—	—	0.736	—
**Q53**	—	—	—	—	—	0.523	—
**Q54**	—	—	—	—	—	—	0.603
**Q55**	—	—	—	—	—	—	0.622
**Q56**	—	—	—	—	—	—	0.718
**Q57**	—	—	—	—	—	—	0.754
**Q58**	—	—	—	—	—	—	0.805
**Q59**	—	—	—	—	—	—	0.764
**Q60**	—	—	—	—	—	—	0.681
**Q61**	—	—	—	—	—	—	0.561
**Factor Loading Range**	**0.772–0.901**	**0.849–0.934**	**0.499–0.864**	**0.669–0.894**	**0.750–0.795**	**0.480–0.921**	**0.561–0.805**

**Table 3:** Proportion of variance explained by each factor (EFA).

Factor	Number of Items	Proportion of Variance (%)
**Perceived Severity**	3	5.6
**Perceived Susceptibility**	4	7.9
**Perceived Benefits**	8	11.9
**Perceived Barriers**	8	13.3
**Self-Efficacy 1**	5	7.9
**Self-Efficacy 2**	5	7.9
**Cues to Action**	8	11
**Total**	41	65.5

**Table 4:** Internal consistency of the HBM subscales.

Factor	Items	Cronbach’s α	RMSR
**Perceived Severity**	3	0.88	0.028
**Perceived Susceptibility**	4	0.935	0.028
**Perceived Benefits**	8	0.911	0.028
**Perceived Barrier**	8	0.94	0.028
**Self-Efficacy 1**	5	0.887	0.028
**Self-Efficacy2**	5	0.896	0.028
**Cues To Action**	8	0.907	0.028

**Fig 1:**
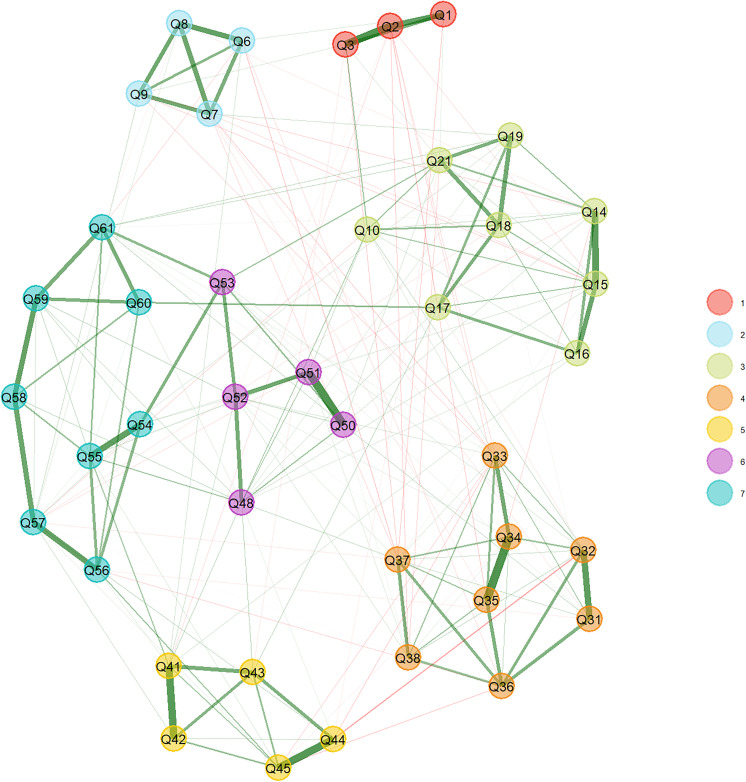
Exploratory Graph Analysis (EGA) of the 41-item HBM scale.

Supplementary analyses further supported the robustness of the factor structure. EGA revealed a seven-community solution that closely aligns with the seven-factor structure identified through EFA. The item clusters correspond well to the theoretical constructs of the HBM, including a two-dimensional structure for self-efficacy and a distinct cluster for cues to action. This structure is visually represented in the EGA network plot (Fig 1), where each color denotes a distinct community.

Nodes represent questionnaire items, and edges indicate regularized partial correlations estimated via the glasso model. Colors indicate community membership.

Table 5 summarizes the overview of Item Response Theory (IRT) results at the dimensional level for the seven HBM constructs. The proportion of variance explained by each dimension ranged from 0.716 to 0.895, indicating good measurement coverage across all subscales. These findings support the reliability and construct validity of the adapted scale. Detailed item-level IRT results, including factor loadings, communalities, discrimination, and difficulty parameters, are provided in S1 Table.

**Table 5:** Overview of IRT results at the dimensional level.

Factor	Items	SS Loadings	Proportion of Variance Explained
**Perceived Severity**	Q1 - Q3	2.482	0.827
**Perceived Susceptibility**	Q6 - Q9	3.581	0.895
**Perceived Benefits**	Q10, Q14 - Q21	5.843	0.730
**Perceived Barriers**	Q31 - Q38	6.36	0.795
**Self-Efficacy1**	Q41 - Q45	3.714	0.743
**Self-Efficacy2**	Q48 - Q53	3.914	0.783
**Cues to action**	Q54 - Q61	5.728	0.716

### Confirmatory Factor Analysis (CFA)

CFA was conducted to test the factor structure identified during EFA. Three models were evaluated based on varying item retention criteria. The final model (Model 3) retained 41 items across seven latent factors, preserving item Q10 due to its theoretical relevance, despite a marginally lower loading.

#### Model fit indices.

Model fit indices indicated good model fit: χ^2^ = 1419.975, df = 753, χ^2^/df = 1.892, CFI = 0.914, TLI = 0.906, RMSEA = 0.059 (90% CI = 0.054–0.064), SRMR = 0.060 (Table 6). The overall model fit indices supported the adequacy of the CFA model. To improve model fit, five residual covariances were added based on the highest modification indices (MIs > 20) and strong theoretical justification [20]. These covariances involved item pairs within the same factor and reflected conceptual similarity or redundancy in item content. Although some additional MI values remained above conventional thresholds, further modifications were not made to avoid overfitting and to preserve theoretical parsimony [19]. The resulting model demonstrated substantially improved fit without compromising the integrity of the factor structure [23].

**Table 6:** Model fit indices for CFA.

Model	χ^2^	df	χ^2^/df	CFI	TLI	RMSEA (90% CI)	SRMR
**CFA Model**	1419.975	753	1.892	0.914	0.906	0.059(0.054-0.064)	0.06

#### Factor loadings.

All standardized factor loadings in the final model were statistically significant (p < 0.001), ranging from 0.526 to 0.936, exceeding the minimum recommended threshold (≥0.50), confirming the robustness of the identified factors (Table 7).

**Table 7:** Standardized factor loadings from CFA.

Item	Factor	Standardized Loadings (Std.all)	SE	p-value
Q1	Perceived Severity	0.797	Fixed	Fixed
Q2	Perceived Severity	0.894	0.081	<0.001
Q3	Perceived Severity	0.838	0.066	<0.001
Q6	Perceived Susceptibility	0.858	Fixed	Fixed
Q7	Perceived Susceptibility	0.887	0.051	<0.001
Q8	Perceived Susceptibility	0.920	0.056	<0.001
Q9	Perceived Susceptibility	0.866	0.058	<0.001
Q10	Perceived Benefits	0.526	Fixed	Fixed
Q14	Perceived Benefits	0.819	0.132	<0.001
Q15	Perceived Benefits	0.826	0.132	<0.001
Q16	Perceived Benefits	0.803	0.131	<0.001
Q17	Perceived Benefits	0.767	0.138	<0.001
Q18	Perceived Benefits	0.860	0.131	<0.001
Q19	Perceived Benefits	0.769	0.139	<0.001
Q21	Perceived Benefits	0.755	0.134	<0.001
Q31	Perceived Barrier	0.698	Fixed	Fixed
Q32	Perceived Barrier	0.755	0.059	<0.001
Q33	Perceived Barrier	0.845	0.109	<0.001
Q34	Perceived Barrier	0.936	0.117	<0.001
Q35	Perceived Barrier	0.930	0.115	<0.001
Q36	Perceived Barrier	0.832	0.078	<0.001
Q37	Perceived Barrier	0.759	0.100	<0.001
Q38	Perceived Barrier	0.676	0.093	<0.001
Q41	Self-Efficacy 1	0.835	Fixed	Fixed
Q42	Self-Efficacy 1	0.833	0.045	<0.001
Q43	Self-Efficacy 1	0.808	0.071	<0.001
Q44	Self-Efficacy 1	0.650	0.084	<0.001
Q45	Self-Efficacy 1	0.703	0.081	<0.001
Q48	Self-Efficacy2	0.773	Fixed	Fixed
Q50	Self-Efficacy2	0.737	0.086	<0.001
Q51	Self-Efficacy2	0.795	0.087	<0.001
Q52	Self-Efficacy2	0.885	0.085	<0.001
Q53	Self-Efficacy2	0.728	0.098	<0.001
Q54	Cues To Action	0.665	Fixed	Fixed
Q55	Cues To Action	0.746	0.069	<0.001
Q56	Cues To Action	0.630	0.086	<0.001
Q57	Cues To Action	0.631	0.103	<0.001
Q58	Cues To Action	0.794	0.098	<0.001
Q59	Cues To Action	0.851	0.101	<0.001
Q60	Cues To Action	0.755	0.104	<0.001
Q61	Cues To Action	0.754	0.103	<0.001

#### Inter-factor correlations.

Inter-factor correlations from the CFA are summarized in Table 8. Notably, Self-Efficacy 1 and Self-Efficacy 2 showed a strong correlation (r = 0.382), and both were highly associated with Cues to Action (r = 0.461 and r = 0.701, respectively), suggesting that individuals who feel more capable are more likely to respond to health prompts or reminders. Similarly, Perceived Benefits was positively related to Self-Efficacy 1 (r = 0.328), Self-Efficacy 2 (r = 0.460), and Cues to Action (r = 0.415), indicating that those who recognize benefits are more confident and receptive to cues. In contrast, Perceived Barriers showed negative correlations with nearly all other factors—most strongly with Perceived Susceptibility (r = −0.373) and Perceived Severity (r = −0.363), suggesting that perceived obstacles reduce the likelihood of recognizing risk or seriousness.

**Table 8:** Inter-factor correlation matrix.

Factor	Perceived Severity	Perceived Susceptibility	Perceived Benefits	Perceived Barrier	Self-Efficacy 1	Self-Efficacy2	Cues To Action
**Perceived Severity**	1.000	0.189	0.028	−0.363	−0.016	−0.049	0.010
**Perceived** **Susceptibility**	0.189	1.000	−0.032	−0.373	0.145	−0.040	0.036
**Perceived Benefits**	0.028	−0.032	1.000	−0.057	0.328	0.460	0.415
**Perceived Barrier**	−0.363	−0.373	−0.057	1.000	−0.247	0.168	−0.031
**Self-Efficacy 1**	−0.016	0.145	0.328	−0.247	1.000	0.382	0.461
**Self-Efficacy2**	−0.049	−0.040	0.460	0.168	0.382	1.000	0.701
**Cues To Action**	0.010	0.036	0.415	−0.031	0.461	0.701	1.000

#### Reliability and convergent validity.

All subscales demonstrated acceptable to excellent reliability, with ω and CR values exceeding the recommended threshold of 0.70 (Table 9). AVE values ranged from 0.526 to 0.779, exceeding the minimum acceptable level of 0.50, thus confirming convergent validity across all seven dimensions of the HBM instrument.

**Table 9:** Reliability and convergent validity.

Subscale	McDonald’s ω	Composite Reliability (CR)	Average Variance Extracted (AVE)
Perceived Severity	0.881	0.881	0.713
Perceived Susceptibility	0.934	0.934	0.779
Perceived Benefits	0.917	0.917	0.576
Perceived Barrier	0.916	0.916	0.659
Self-Efficacy 1	0.851	0.851	0.595
Self-Efficacy2	0.869	0.869	0.621
Cues To Action	0.868	0.868	0.526

**Fig 2:**
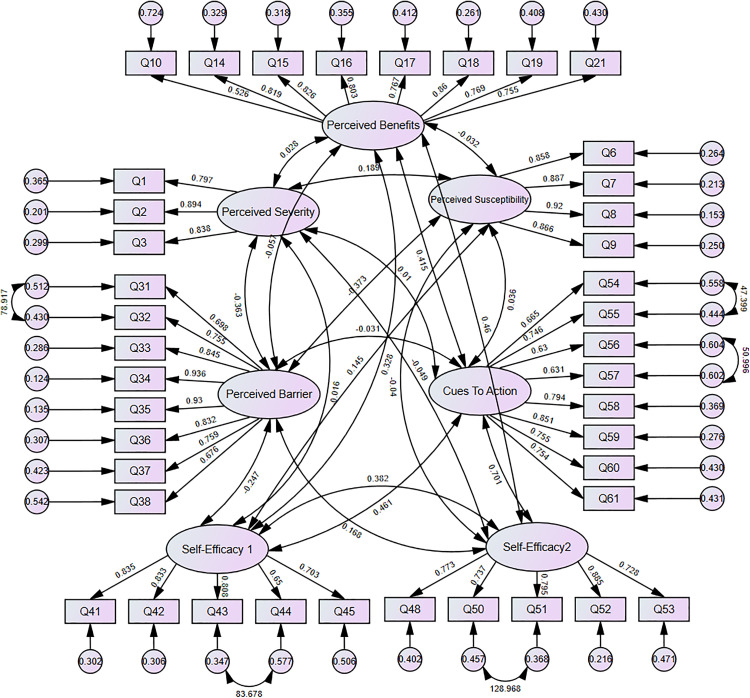
Confirmatory factor analysis model for the measurement structure of latent constructs.

A conceptual path diagram illustrating the final CFA model is presented in Fig 2.

This diagram illustrates the confirmatory factor analysis (CFA) model of the final 41-item Health Belief Model (HBM) questionnaire for breast cancer screening. Ovals represent latent constructs, including perceived susceptibility, perceived severity, perceived benefits, perceived barriers, cues to action, and two empirically distinct self-efficacy dimensions. Rectangles represent observed items (Q1–Q61). Solid arrows denote standardized factor loadings of items on their respective latent variables, while double-headed arrows indicate inter-factor correlations. Numbers on single-headed arrows indicate standardized loadings; numbers on double-headed arrows represent correlations. Numbers inside small circles indicate residual variances. Five additional residual covariances (arrows between item residuals) were added based on high modification indices to improve model fit. These were theoretically justifiable due to item content overlap.

### Measurement Invariance (MI)

MI was assessed across two key demographic subgroups: residential settings (urban vs. non-urban) and age groups (18–40 vs. 41–70 years) using multi-group CFA. Four nested models were tested—configural, metric, scalar, and strict invariance—and model fit was evaluated via CFI, RMSEA, and SRMR.

For residential settings, all ΔCFI (≤ 0.001), ΔRMSEA (≤ 0.001), and ΔSRMR (≤ 0.001) values remained within acceptable thresholds (Table 10), indicating full measurement invariance. Similarly, across age groups, fit differences between nested models were minimal (ΔCFI ≤ 0.004; ΔRMSEA ≤ 0.001; ΔSRMR ≤ 0.002), supporting factorial equivalence between younger and older participants (Table 11).

**Table 10:** Measurement invariance across residential settings.

Model	χ^2^ (df)	CFI	ΔCFI	RMSEA	ΔRMSEA	SRMR	ΔSRMR
**Configural**	1.885	0.857	—	0.078	—	0.069	—
**Metric**	1.867	0.857	0.000	0.078	0.000	0.070	0.001
**Scalar**	1.850	0.857	0.000	0.077	−0.001	0.071	0.001
**Strict**	1.787	0.858	0.001	0.076	−0.001	0.070	− 0.001

Note: Measurement invariance was supported across residential settings, with all ΔCFI, ΔRMSEA, and ΔSRMR values falling within acceptable thresholds (|ΔCFI| ≤ 0.01, |ΔRMSEA| ≤ 0.015, |ΔSRMR| ≤ 0.03).

**Table 11:** Measurement invariance across groups (Age).

Model	χ^2^ (df)	CFI	ΔCFI	RMSEA	ΔRMSEA	SRMR	ΔSRMR
**Configural**	1.906	0.853	—	0.080	—	0.074	—
**Metric**	1.896	0.852	−0.001	0.079	−0.001	0.076	0.002
**Scalar**	1.874	0.852	0.000	0.078	−0.001	0.077	0.001
**Strict**	1.850	0.848	−0.004	0.079	0.001	0.077	0.000

Note: Measurement invariance was supported across age groups, with all ΔCFI, ΔRMSEA, and ΔSRMR values falling within acceptable thresholds (|ΔCFI| ≤ 0.01, |ΔRMSEA| ≤ 0.015, |ΔSRMR| ≤ 0.03).

## Discussion

### Structural validity and reliability

The final CFA model supported a seven-factor solution that reflected the six theoretical constructs of the HBM, with the self-efficacy dimension empirically splitting into two distinct but conceptually coherent components. Compared with HBM validation studies conducted in countries such as America [24], Egypt [25], Ethiopia [26], Iran [27], the seven-factor solution observed in this study demonstrates strong theoretical alignment and cultural adaptability within the Chinese context. The structural distinction observed in self-efficacy reflects the multidimensional nature of this construct within the context of BCS. The split revealed two psychologically distinct components: confidence in performing breast self-examination techniques, and the perceived ability to manage emotional uncertainty, information needs, and practical challenges such as scheduling or cost. These dimensions were reflected in retained items such as confidence in using BSE techniques, and self-reliant coping with scheduling and financial barriers, highlighting an internalized form of self-efficacy. This specific structure may be shaped by sociocultural patterns unique to Chinese women, where health decisions are often made independently due to cultural values that emphasize personal responsibility, emotional restraint, and avoidance of burdening others. These norms, combined with limited access to structured screening systems, may lead women to rely on personal coping and logistical strategies when facing screening-related decisions. Notably, similar distinctions in the multidimensional nature of self-efficacy have been reported in other validation studies. For instance, Diotaiuti et al. identified factorial complexity in the domain of self-regulatory behavior, which supports the idea that self-efficacy may manifest through distinct yet interrelated psychological dimensions depending on context [28]. Such differentiation provides theoretical insight for developing culturally sensitive screening interventions that align with women’s psychological profiles and lived experiences. In addition, the finalized 41-item version of the instrument achieved an appropriate balance between comprehensiveness and practicality. While previous HBM tools have varied widely in item quantity and domain representation, the current version is both theory-driven and empirically optimized. It offers comprehensive coverage of core HBM constructs while maintaining a manageable length suitable for public health surveys and routine clinical use.

Among the three CFA models evaluated, Model 3 was ultimately selected based on a balanced consideration of model fit, parsimony, and theoretical alignment. Model 1 required the removal of 14 items but necessitated the addition of at least 10 residual covariances to achieve acceptable fit, raising concerns about potential overfitting. Model 2 demonstrated acceptable fit with only four residual covariances but excluded item Q10“Performing BSE monthly helps in early detection of breast cancer”, which was considered theoretically essential and endorsed by expert consensus. In the final model, Q10 showed a standardized factor loading of 0.526, exceeding the commonly accepted threshold (≥ 0.50), indicating satisfactory performance. This item, categorized under the Perceived Benefits dimension, directly reflects women’s belief in the important benefit of early breast cancer detection through monthly breast self-examination (BSE) and serves as a key psychological driver of screening behavior. Although its factor loading is slightly lower compared to some other items, it exceeds the accepted standard and contributes significantly to the measurement of the construct. The overall model fit remains good, and experts agree that retaining Q10 is critical for fully capturing the Perceived Benefits dimension. Removing this item could compromise the content validity and structural integrity of the scale. Model 3 retained Q10, required only five residual covariances, and demonstrated comparable fit indices, making it the most parsimonious and theoretically coherent solution.

### Measurement invariance across subgroups

MI testing confirmed that the questionnaire measures the same underlying constructs across key demographic subgroups, supporting valid comparisons of latent means. Notably, this study is among the few to assess measurement equivalence of an HBM instrument in a Chinese context, addressing a gap in cross-cultural validation research.

While the final CFA model showed good fit (CFI = 0.914), CFI values for invariance models across residential and age groups were slightly lower (0.848–0.858), likely due to increasing model constraints limiting flexibility to capture group-specific variation [22]. Model complexity, including the number of items and factors, affects various fit indices; more parsimonious models impose stricter constraints, which may lead to slight decreases in fit indices but enhance model interpretability and generalizability. This trade-off is important when interpreting measurement invariance results [29]. Importantly, despite the decline in absolute CFI values, the minimal changes in CFI (ΔCFI ≤ 0.01) and RMSEA (ΔRMSEA ≤ 0.015) fall well within accepted thresholds for measurement invariance, supporting meaningful comparisons across subgroups [30].

### Latent construct relationships

The interrelationships observed among HBM constructs provided both theoretical and practical insight. Notably, strong associations were observed between self-efficacy, cues to action, and perceived benefits. This suggests that women who feel more capable are also more attentive to screening prompts and better appreciate the value of early detection [31,32]. In contrast, perceived barriers appeared to diminish risk perception and concern, consistent with their role as psychological or logistical deterrents to screening engagement [33,34].

These patterns reinforce the theoretical logic of HBM, where health behavior is influenced by a dynamic interplay between motivation, perceived control, and external stimuli [35]. Rather than functioning in isolation, the constructs interact in a way that reflects decision-making processes [36,37]. Recognizing these interactions is essential for designing targeted interventions. For example, boosting self-efficacy could simultaneously enhance response to cues and amplify perceived screening benefits, thereby improving participation rates [38].

### Implications for practice

Based on observed inter-construct relationships, interventions can be created as either a single-construct or a multi-construct approach. A single-construct intervention focuses on addressing one specific psychological construct at a time. For example, women reporting high levels of perceived barriers may benefit from interventions such as private examination spaces, personalized counseling, and educational materials addressing discomfort, inconvenience, and cost [39]. Women with low levels of self-efficacy may benefit from interventions such as skill-building workshops, online videos, and live demonstrations to enhance confidence and technique [40]. Similarly, for women who perceive fewer benefits of screening, may benefit from getting authentic patient stories which shows the benefits of early detection, improving their perceived value [41]. Automated reminders via SMS, mobile apps, or appointment systems can serve as effective intervention methods for addressing low levels of cues to action. Above examples showcases a single-construct intervention approach as they target a single psychological construct such as perceived barriers, self-efficacy, perceived benefits, or cue to action.

Multi-construct intervention approach simultaneously targets a combination of related psychological constructs. Because there is a strong correlation between self-efficacy, perceived benefits, and cues to action, multi-construct strategies may result in a greater behavioral effect. For example, community health events containing skills training, patient testimonials, and on-site scheduling can address multiple psychological constructs at once. Digital platforms that incorporate educational resources, personal narratives, and automated reminders may further enhance this effect [24]. Multi-construct intervention also works for constructs that have a negative association, such as perceived barriers and susceptibility. Interventions such as affordable screening and transportation assistance combined with risk consultations could simultaneously reduce perceived barriers and increase susceptibility. By increasing access, visibility, and perceived personal relevance of screening services, these interventions may also indirectly enhance women’s awareness of their own breast cancer risk [42]. In addition to their psychological impact, multi-construct strategies offer practical benefits for healthcare systems. Especially in rural settings, where access to medical services is limited and follow-up costs are high, an intervention tool that uses multi-construct approach may be especially efficient. It could reduce the need for repeated in-person counseling while reaching larger populations. Health authorities could integrate single-construct and multi-construct approaches into existing public health programs and incorporating various interventions into routine services performed by community health workers.

Taken together, both single- and multi-construct intervention strategies informed by the HBM offer flexible options for tailoring screening promotion efforts to diverse needs and settings. These interventions could be applied systematically across different provinces to address disparities in screening participation. When paired with real-time data tracking and outcomes evaluation, their implementation may become even more effective and sustainable. Importantly, these strategies are grounded in the Chinese context, developed in response to specific patterns of health beliefs and psychological constructs observed among Chinese women. These patterns are shaped by sociocultural norms, educational background, and access to healthcare, and they reflect culturally embedded factors that influence breast cancer screening behavior. Given that Chinese immigrant women around the globe often face similar cultural beliefs and structural challenges in accessing BCS, these HBM interventions may offer valuable guidance for designing culturally responsive screening efforts in other settings as well.

### Limitations and future directions

Several points merit consideration, although the HBM instrument demonstrated strong psychometric properties. First, while adding a few theoretically justified residual covariances improved model fit, further optimization may still be possible [20]. Second, due to sample size limitations (N = 314), exploratory and confirmatory factor analyses were conducted on the full dataset. The original study plan involved confirmatory validation of a theoretically derived structure; however, inadequate model fit indicated structural instability. Therefore, EFA was performed to empirically refine the factor structure, followed by CFA to validate the revised model within the same dataset. While this sequential approach is not optimal, it is widely accepted in initial psychometric validation studies, particularly during early-stage cross-cultural adaptation when independent samples are unavailable [17]. Ideally, EFA and CFA should be conducted on separate subsamples; however, due to data limitations, this was not feasible. It should be noted that conducting EFA and CFA on the same dataset may introduce some risk of model overfitting and somewhat limit the generalizability of the factor structure, potentially impacting model stability and estimation. Future research may consider replicating these findings using independent samples to further strengthen the evidence for structural robustness and generalizability. Third, potential clustering bias may arise from geographic sampling, as participants were recruited from different cities and districts. Variations in socioeconomic factors, healthcare accessibility, and cultural norms across these regions may influence the latent structure of the measured constructs. Future studies should account for this clustering effect by employing appropriate multilevel or cluster-adjusted analyses to further validate the structural robustness of the scale. Fourth, potential selection bias may exist due to the online data collection method, which could exclude women with low digital literacy or limited internet access. This limitation may affect the generalizability of the findings and should be addressed in future studies by employing more inclusive sampling methods. Fifth, the external validity of this study is limited to Guizhou Province. To enhance generalizability, future research should replicate the validation process in other Chinese regions, such as coastal areas, where demographic, socioeconomic, and healthcare contexts differ. Such studies will help determine the applicability of the instrument across diverse populations.

Moreover, subsequent research should evaluate the longitudinal stability and predictive validity of the instrument, with particular attention to its ability to track changes in screening behavior over time or in response to interventions. Although predictive validity was not assessed in the present study, follow-up structural equation modeling (SEM) analyses are currently underway to determine whether the validated HBM constructs can predict actual screening behaviors. Such analyses would provide important evidence of intervention effectiveness and further verify the instrument’s sensitivity to behavioral change. It is also important to note that the current study relied solely on self-reported perceptions, without measuring actual screening behaviors. Therefore, future studies incorporating behavioral follow-up data are warranted to more comprehensively validate the predictive capacity of the instrument in real-world contexts.

## Conclusion

This study validated a culturally adapted instrument assessing Chinese women’s health beliefs about BCS. A robust seven-factor structure was confirmed, reflecting distinct but related dimensions of beliefs influencing screening behaviors. The measurement invariance established between urban and non-urban populations supports the tool’s broad applicability.

Notably, the bifurcation of self-efficacy reflects culturally grounded aspects of screening-related agency. Relationships among the constructs were consistent with theoretical expectations, specifically positive associations between cues to action, perceived benefits, and self-efficacy, and negative associations between perceived barriers and risk perception.

Overall, this instrument provides researchers and practitioners with a reliable and culturally sensitive means to assess screening beliefs, identify barriers, and develop targeted interventions. Future research should examine the instrument’s predictive validity across diverse populations and consider integrating it into comprehensive multi-level models that address psychological, cultural, and structural determinants of breast cancer screening behavior.

## Supporting information

S1 File English version of the final adapted HBM questionnaire used in this study. (PDF)

S1 Dataset File containing the de-identified data used for exploratory and confirmatory factor analysis in this study. (XLSX)

S1 Table IRT factor loadings, communalities (h^2^), and explained variance for each HBM subscale. (DOCX)
