# Supplementary material for: Psychometric validation of a culturally adapted health belief model scale for breast cancer screening in Chinese women
Source: PLoS One. 2025 Sep 3;20(9):e0331279. doi: 10.1371/journal.pone.0331279 (PMC12407439; doi:10.1371/journal.pone.0331279)
Supplement: S1 File — (PDF) [file pone.0331279.s001.pdf]

Supplementary File 1. English Version of the Adapted HBM Questionnaire

A Survey on Women's Understanding and Attitudes Toward Breast Cancer and Screening

Study ID:

Thank you for participating in this study.

This study will ask about your knowledge and views on participating in breast cancer screening. It also covers topics such as your health and lifestyle. Additionally, it will ask for some information about you.

Protocol code USM/JEPeM/KK/24010004

School of Medical Sciences

University Sains Malaysia

How to fill in this questionnaire

- To answer the questions, please check the appropriate box.
- Don’t spend too much time on each question—your first answer may be the best one. There are no right or wrong answers.
- If you’re unsure how to answer a question, please give the best answer you can.

- You may want to take a break while completing the survey. The information you provide will be kept strictly confidential.

Part 1：Personal Information

1. Age:____________

2. Nationality:
   - What is your nationality?
     - Han Chinese
     - Ethnic minority (please specify)___________

3. What is your current marital status?
   - Single
   - Married
   - Divorced
   - Widowed

4. What is your highest level of education?
   - Primary school or below
   - Junior high school
   - High school/vocational school
   - Associate degree
   - Bachelor's degree
   - Master's degree or above

5. What is your current occupation?
   - Government employee(administrative, public institution staff)
   - Corporate employee

- Business and service industry personnel
- Freelancer
- Student
- Retired
- Hired personnel
- Other (please specify)______________

6. What is your monthly income?
   - Below 2000 RMB
   - 2001--4000 RMB
   - 4001--6000 RMB
   - 6001--8000 RMB
   - 8001-10000 RMB
   - above 10001 RMB

7. How many members are in your family?
   - 1 person
   - 2 persons
   - 3-4 persons
   - 5 persons and above

8. Where do you currently live?
   - Urban area
   - Suburban area
   - Rural area

9. What is your current housing situation?
   - Own house
   - Renting
   - Living with parents

10. Do you have any of the following chronic diseases?

- Hypertension
- Diabetes
- Heart disease
- Cancer
- Other (please specify)___________
- None

11. What is your medical payment method?

- Publicly funded medical care
- urban medical insurance
- Self-funded commercial insurance
- Fully self-funded

12. Has any of your first-degree relatives mother, sister or daughter ever had breast cancer?

- Yes
- No

13. At what age did you have your first menstrual period?_____________

Part 2: Breast Cancer Screening Scale (Health Belief Model)

Please read the statements below carefully and rate how much you agree or disagree with the statement by ticking a box to the right.

Section A: Perceived Severity

1.	When I think about breast	Strongly agree	Agree	neither agree nor disagree	Disagree	strongly disagree

	cancer, my heart beats faster					
2.	I am afraid even to think about breast cancer	Strongly agree	Agree	neither agree nor disagree	Disagree	strongly disagree
3.	The thought of breast cancer scares me	Strongly agree	Agree	neither agree nor disagree	Disagree	strongly disagree
4.	I think I will not survive more than 5 years if I have breast cancer	Strongly agree	Agree	neither agree nor disagree	Disagree	strongly disagree
5.	All my life will be changed if I got breast cancer	Strongly agree	Agree	neither agree nor disagree	Disagree	strongly disagree

Section B: Perceived Susceptibility

6.	I am susceptible to breast cancer in the future	Strongly agree	Agree	neither agree nor disagree	Disagree	strongly disagree
7.	I think that I am more susceptible to get breast cancer than other people	Strongly agree	Agree	neither agree nor disagree	Disagree	strongly disagree
8.	My personal chance of getting breast cancer	Strongly agree	Agree	neither agree nor disagree	Disagree	strongly disagree

	is high					
9.	I am highly susceptible to breast cancer in next 10 years	Strongly agree	Agree	neither agree nor disagree	Disagree	strongly disagree

Section C: Perceived Benefits

10.	Performing BSE monthly help in early detection of breast cancer	Strongly agree	Agree	neither agree nor disagree	Disagree	strongly disagree
11.	Performing BSE monthly help in detection of tumors before going to the doctors	Strongly agree	Agree	neither agree nor disagree	Disagree	strongly disagree
12.	Performing BSE monthly will decrease complications of breast cancer if I got breast cancer	Strongly agree	Agree	neither agree nor disagree	Disagree	strongly disagree
13.	Performing BSE decrease the anxiety about breast cancer	Strongly agree	Agree	neither agree nor disagree	Disagree	strongly disagree
14.	Mammogram is effective for the early detection of breast cancer.	Strongly agree	Agree	neither agree nor disagree	Disagree	strongly disagree
15.	Mammogram can help in detecting lumps that might be missed by doctors.	Strongly agree	Agree	neither agree nor disagree	Disagree	strongly disagree
16.	Mammogram is more effective than clinician or	Strongly agree	Agree	neither agree nor disagree	Disagree	strongly disagree

	breast selfexamination					
17.	When I get a mammogram, I do not worry as much about breast cancer	Strongly agree	Agree	neither agree nor disagree	Disagree	strongly disagree
18.	Having a mammogram will help me find lumps early in my breasts	Strongly agree	Agree	neither agree nor disagree	Disagree	strongly disagree
19.	If I find a lump through a mammogram, the treatment for breast cancer may not be as bad	Strongly agree	Agree	neither agree nor disagree	Disagree	strongly disagree
20.	Having a mammogram will decrease my chances of dying from breast cancer	Strongly agree	Agree	neither agree nor disagree	Disagree	strongly disagree
21.	Having a mammogram will help me find a lump before it can be felt by myself or a health professional	Strongly agree	Agree	neither agree nor disagree	Disagree	strongly disagree
22.	Keeping good health is important to me.	Strongly agree	Agree	neither agree nor disagree	Disagree	strongly disagree
23.	I wish to discover health problems that occur early.	Strongly agree	Agree	neither agree nor disagree	Disagree	strongly disagree
24.	I consume a balanced diet.	Strongly agree	Agree	neither agree nor disagree	Disagree	strongly disagree
25.	I exercise at	Strongly	Agree	neither	Disagree	strongly

	least 3 times a week.	agree		agree nor disagree		disagree
26.	I perform annual medical check-up.	Strongly agree	Agree	neither agree nor disagree	Disagree	strongly disagree

Section D: Perceived Barriers

27.	Performing BSE is time consuming	Strongly agree	Agree	neither agree nor disagree	Disagree	strongly disagree
28.	Performing BSE is tedious	Strongly agree	Agree	neither agree nor disagree	Disagree	strongly disagree
29.	Having a mammogram would take too much time	Strongly agree	Agree	neither agree nor disagree	Disagree	strongly disagree
30.	Screening mammogram is difficult to fit into my schedule	Strongly agree	Agree	neither agree nor disagree	Disagree	strongly disagree
31.	Performing BSE is unpleasant to me	Strongly agree	Agree	neither agree nor disagree	Disagree	strongly disagree
32.	Feeling of shame and embarrassment when performing BSE	Strongly agree	Agree	neither agree nor disagree	Disagree	strongly disagree
33.	Performing BSE increase my anxiety about having breast	Strongly agree	Agree	neither agree nor disagree	Disagree	strongly disagree
34.	Having a routine mammogram would make me anxious about breast cancer	Strongly agree	Agree	neither agree nor disagree	Disagree	strongly disagree

35.	Having a routine mammogram would make me worry	Strongly agree	Agree	neither agree nor disagree	Disagree	strongly disagree
36.	Having a mammogram would be embarrassing	Strongly agree	Agree	neither agree nor disagree	Disagree	strongly disagree
37.	Having a mammogram would be painful	Strongly agree	Agree	neither agree nor disagree	Disagree	strongly disagree
38.	Having a mammogram would cost too much	Strongly agree	Agree	neither agree nor disagree	Disagree	strongly disagree
39.	I think getting breast cancer is fated and BSE will not change it	Strongly agree	Agree	neither agree nor disagree	Disagree	strongly disagree
40.	No suitable place at home to perform BSE	Strongly agree	Agree	neither agree nor disagree	Disagree	strongly disagree

Section E: Self- efficacy

41.	I am confident in performing BSE correctly	Strongly agree	Agree	neither agree nor disagree	Disagree	strongly disagree
42.	I can use the pads of my fingers correctly when performing BSE.	Strongly agree	Agree	neither agree nor disagree	Disagree	strongly disagree
43.	I am confident I can discover breast tumours by performing BSE	Strongly agree	Agree	neither agree nor disagree	Disagree	strongly disagree
44.	I can discover breast tumor at	Strongly agree	Agree	neither agree nor	Disagree	strongly disagree

	size of small pea			disagree		
45.	I am able to differentiate between normal and abnormal breast tissue through BSE	Strongly agree	Agree	neither agree nor disagree	Disagree	strongly disagree
46.	I can make an appointment for a mammogram	Strongly agree	Agree	neither agree nor disagree	Disagree	strongly disagree
47.	I can arrange transportation to get a mammogram	Strongly agree	Agree	neither agree nor disagree	Disagree	strongly disagree
48.	I can arrange my schedule to have a mammogram	Strongly agree	Agree	neither agree nor disagree	Disagree	strongly disagree
49.	I can talk to other people at the mammogram center about my concerns	Strongly agree	Agree	neither agree nor disagree	Disagree	strongly disagree
50.	I can get a mammogram even if I am worried	Strongly agree	Agree	neither agree nor disagree	Disagree	strongly disagree
51.	I can get a mammogram even if I don’t know what to expect	Strongly agree	Agree	neither agree nor disagree	Disagree	strongly disagree
52.	I can find a way to pay for a mammogram	Strongly agree	Agree	neither agree nor disagree	Disagree	strongly disagree
53.	I always seek new information that improves my health.	Strongly agree	Agree	neither agree nor disagree	Disagree	strongly disagree

Section F: Cues to action

54.	I have heard about BSE from the mass media	Strongly agree	Agree	neither agree nor disagree	Disagree	strongly disagree
55.	I have heard about BSE from talking with people in my social and medical circles.	Strongly agree	Agree	neither agree nor disagree	Disagree	strongly disagree
56.	Knowing someone who had breast cancer motivated me to perform BSE	Strongly agree	Agree	neither agree nor disagree	Disagree	strongly disagree
57.	Having a close relative who had breast cancer made me do BSE	Strongly agree	Agree	neither agree nor disagree	Disagree	strongly disagree
58.	Reminder letters would help me to get a mammogram	Strongly agree	Agree	neither agree nor disagree	Disagree	strongly disagree
59.	Routine educational talks regarding breast cancer awareness would help me to get a mammogram	Strongly agree	Agree	neither agree nor disagree	Disagree	strongly disagree
60.	I feel confident that if I had a mammogram done, any abnormalities	Strongly agree	Agree	neither agree nor disagree	Disagree	strongly disagree

	in my breasts will be detected					
61.	In case I need a mammogram, I will find a place to get it done	Strongly agree	Agree	neither agree nor disagree	Disagree	strongly disagree

Thank you for your participation.
