# Supplementary material for: Psychometric validation of a culturally adapted health belief model scale for breast cancer screening in Chinese women
Source: PLoS One. 2025 Sep 3;20(9):e0331279. doi: 10.1371/journal.pone.0331279 (PMC12407439; doi:10.1371/journal.pone.0331279)
Supplement: S1 Table — (DOCX) [file pone.0331279.s003.docx]

S1 Table. IRT factor loadings, communalities (h²), and explained variance for each HBM subscale

| **Subscale** | **Item** | **Factor Loading** | **Communality (h²)** |
| --- | --- | --- | --- |
| Perceived Severity | Q1 | 0.858 | 0.736 |
| Perceived Severity | Q2 | 0.954 | 0.910 |
| Perceived Severity | Q3 | 0.914 | 0.835 |
| Perceived Susceptibility | Q6 | 0.928 | 0.861 |
| Perceived Susceptibility | Q7 | 0.949 | 0.900 |
| Perceived Susceptibility | Q8 | 0.970 | 0.940 |
| Perceived Susceptibility | Q9 | 0.937 | 0.878 |
| Perceived Benefits | Q10 | 0.661 | 0.437 |
| Perceived Benefits | Q14 | 0.894 | 0.798 |
| Perceived Benefits | Q15 | 0.903 | 0.816 |
| Perceived Benefits | Q16 | 0.862 | 0.743 |
| Perceived Benefits | Q17 | 0.842 | 0.708 |
| Perceived Benefits | Q18 | 0.932 | 0.869 |
| Perceived Benefits | Q19 | 0.868 | 0.753 |
| Perceived Benefits | Q21 | 0.847 | 0.717 |
| Perceived Barriers | Q31 | 0.827 | 0.685 |
| Perceived Barriers | Q32 | 0.869 | 0.755 |
| Perceived Barriers | Q33 | 0.910 | 0.828 |
| Perceived Barriers | Q34 | 0.967 | 0.935 |
| Perceived Barriers | Q35 | 0.964 | 0.930 |
| Perceived Barriers | Q36 | 0.921 | 0.847 |
| Perceived Barriers | Q37 | 0.854 | 0.729 |
| Perceived Barriers | Q38 | 0.807 | 0.652 |
| Self-Efficacy1 | Q41 | 0.873 | 0.762 |
| Self-Efficacy1 | Q42 | 0.890 | 0.792 |
| Self-Efficacy1 | Q43 | 0.897 | 0.805 |
| Self-Efficacy1 | Q44 | 0.806 | 0.650 |
| Self-Efficacy1 | Q45 | 0.839 | 0.703 |
| Self-Efficacy2 | Q48 | 0.820 | 0.672 |
| Self-Efficacy2 | Q50 | 0.944 | 0.891 |
| Self-Efficacy2 | Q51 | 0.968 | 0.938 |
| Self-Efficacy2 | Q52 | 0.883 | 0.780 |
| Self-Efficacy2 | Q53 | 0.796 | 0.634 |
| Cues to action | Q54 | 0.827 | 0.684 |
| Cues to action | Q55 | 0.884 | 0.781 |
| Cues to action | Q56 | 0.817 | 0.667 |
| Cues to action | Q57 | 0.790 | 0.624 |
| Cues to action | Q58 | 0.892 | 0.795 |
| Cues to action | Q59 | 0.896 | 0.804 |
| Cues to action | Q60 | 0.840 | 0.706 |
| Cues to action | Q61 | 0.817 | 0.667 |

Note: All IRT models were fitted using the mirt package (graded response model). Proportion of variance explained for each factor ranged from 0.716 to 0.895.
